# Lactide: Production Routes, Properties, and Applications

**DOI:** 10.3390/bioengineering9040164

**Published:** 2022-04-07

**Authors:** Bruna L. C. Cunha, Juliana O. Bahú, Letícia F. Xavier, Sara Crivellin, Samuel D. A. de Souza, Leandro Lodi, André L. Jardini, Rubens Maciel Filho, Maria I. R. B. Schiavon, Viktor O. Cárdenas Concha, Patricia Severino, Eliana B. Souto

**Affiliations:** 1 School of Chemical Engineering, Federal University of São Paulo (UNIFESP), Diadema 09913-030, São Paulo, Brazil; b.carvalhocunha@hotmail.com (B.L.C.C.); leticia.funari@hotmail.com (L.F.X.); viktor.cardenas@unifesp.br (V.O.C.C.); 2 School of Chemical Engineering, University of Campinas (UNICAMP), Campinas 13083-852, São Paulo, Brazil; saracrivellin19@gmail.com (S.C.); samuel.diogenes.pgfeq@gmail.com (S.D.A.d.S.); ajardini@unicamp.br (A.L.J.); rmaciel@unicamp.br (R.M.F.); ingridrb@unicamp.br (M.I.R.B.S.); 3 Institute of Science and Technology, Federal University of Alfenas (UNIFAL), Poços de Caldas 37715-400, Minas Gerais, Brazil; leandro.lodi@unifal-mg.edu.br; 4 Laboratory of Nanotechnology and Nanomedicine (LNMed), Institute of Technology and Research (ITP), Av. Murilo Dantas, 300, Aracaju 49010-390, Sergipe, Brazil; patricia_severino@itp.org.br; 5 Industrial Biotechnology Program, University of Tiradentes (UNIT), Av. Murilo Dantas, 300, Aracaju 49010-390, Sergipe, Brazil; 6 Department of Pharmaceutical Technology, Faculty of Pharmacy, University of Porto, Rua de Jorge Viterbo Ferreira, 228, 4050-313 Porto, Portugal; 7 REQUIMTE/UCIBIO, Faculty of Pharmacy, University of Porto, Rua de Jorge Viterbo Ferreira, 228, 4050-313 Porto, Portugal

**Keywords:** applications, industrial processes, l-lactide, market costs, synthesis

## Abstract

Lactide dimer is an important monomer produced from lactic acid dehydration, followed by the prepolymer depolymerization process, and subsequent purification. As lactic acid is a chiral molecule, lactide can exist in three isomeric forms: L-, D-, and *meso*-lactide. Due to its time-consuming synthesis and the need for strict temperature and pressure control, catalyst use, low selectivity, high energy cost, and racemization, the value of a high purity lactide has a high cost in the market; moreover, little is found in scientific articles about the monomer synthesis. Lactide use is mainly for the synthesis of high molar mass poly(lactic acid) (PLA), applied as bio-based material for medical applications (e.g., prostheses and membranes), drug delivery, and hydrogels, or combined with other polymers for applications in packaging. This review elucidates the configurations and conditions of syntheses mapped for lactide production, the main properties of each of the isomeric forms, its industrial production, as well as the main applications in the market.

## 1. Introduction

Lactide (3,6-dimethyl-1,4-dioxane-2,5-dione) is a cyclic ester used as an intermediate product in the production of poly(lactic acid) (PLA), the desired monomer for high-molar mass PLA synthesis. This dimer has a high cost in the market, due to its expensive and time-consuming process for its synthesis. Factors such as racemization, low selectivity, and high energy consumption are associated with its production procedure [1]. Low molar mass PLA produces lactide, which is produced via depolymerization involving back-biting and end-biting reactions. In general, the back-biting reaction is an intramolecular reaction between the carboxylic terminal group and the ester bond, while end-biting refers to the ring closure of the chain [2]. The price increase of lactide (Table 1) translates into its commercial interest.

**Figure 1:**
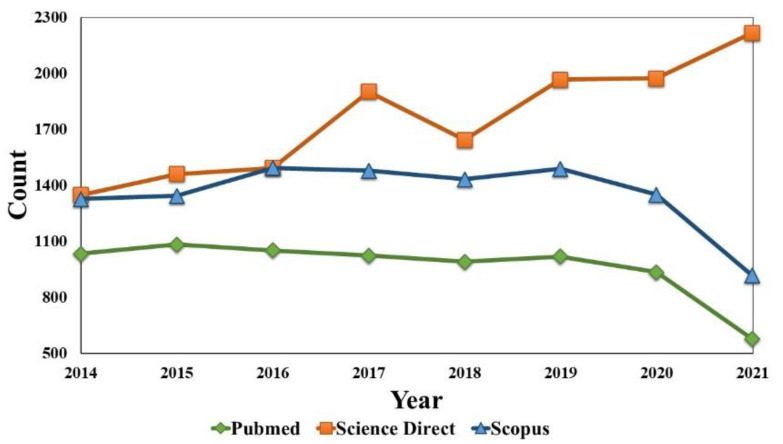
The number of publications about lactide in the last few years in common database research platforms for the search item “lactide”.

The lactide purity depends on the reaction conditions and the oligomer composition; therefore, both factors must be considered in the dimer production. Temperature, time, pressure, oligomer molecular weight, as well as the type and concentration of catalyst, are parameters that must be optimized to achieve a high yield of lactide with a low degree of racemization [2]. Once derived from lactic acid, which is a chiral molecule, the lactide has two stereocenters, existing as two enantiomers (L- and D-lactide, melting point (MP): 97 °C), besides its *meso* (MP: 53 °C) and racemic mixture (MP: 125 °C) [2]. The interest in its L- over D- isomer is related to the bioabsorbable and biocompatible characteristics of L-lactide, which are required for biomaterials to be metabolized by mammals [3]. The relevance of this product on the market, combined with its growing demand, is high [4]. A reasonable amount of work on the lactide, a monomer used in the PLA synthesis by ring-opening polymerization (ROP), can be seen in Figure 1. However, little information is found about the synthesis and processes for obtaining the lactide, with the majority of work related to its production found in patents. In this sense, a joint effort between industry and academia is necessary to fill this gap [5]. According to this, the present review presents aspects of lactide chain production and synthesis peculiarities. Here can also be found its applications, specifically in the medical area, in addition to some market considerations.

## 2. Synthesis

Pure lactic acid after fermentation of carbohydrates is required to produce lactide, because impurities (alcohols, carboxylic acids, etc.) in this process can negatively impact the lactide formation, which is also mandatory for a clean procedure to further produce high-quality polymers, especially for the medical area [6,7,8,9,10].

**Figure 2:**
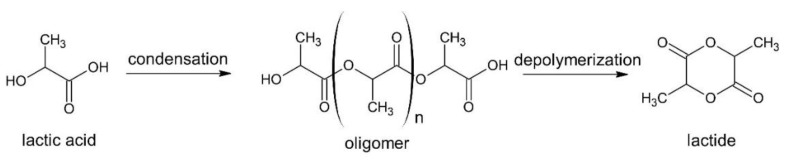
Lactide dimer synthesis (reprinted with permission [12], Copyright 1998 Elsevier).

The lactide is a cyclic ester formed by two lactic acid molecules obtained via lactic acid polycondensation, followed by depolymerization of the PLA prepolymer under heating and reduced pressure (Figure 2) [11].

The lactide formation occurs in two steps: first, a prepolymer (oligomer) is obtained by lactic acid dehydration, then the lactide is synthesized through a depolymerization process. Generally, both steps take place at elevated temperatures (>200 °C), which implicates high energy consumption. In addition, the purification of the lactide is a crucial step that can lead to a cost increase in the production of PLA via ROP [13].

**Figure 3:**
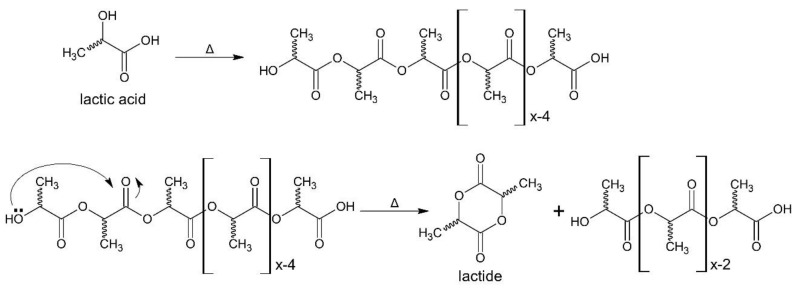
Back-biting reaction of the OH group in the PLA chain (reprinted with permission [15], Copyright 2004 Elsevier).

Another route for lactide production is using reverse engineering, that is, the depolymerization of the oligomeric PLA in reactions known as back-biting and end-biting. The back-biting reaction yields a cyclic compound via intramolecular reactions between the hydroxylic and the ester group of this oligomer chain (Figure 3) [14].

These are equilibrium reactions, dependent on the temperature and size of the cyclic compound. The “end-biting” reaction closes the linear chain to produce a lactide ring and water, while the “back-biting” produces cyclic and linear components [14]. Concerning the yield, the main factors that affect the lactide yield from the lactic acid oligomer are [16]:

- Temperature, since the reaction rate, the vapor pressure of lactide from the reaction mixture, and the rate of racemization of lactide are proportional to heating;
- Pressure is inversely proportional to the depolymerization rate of the prepolymer and lactide yield.

**Figure 4:**
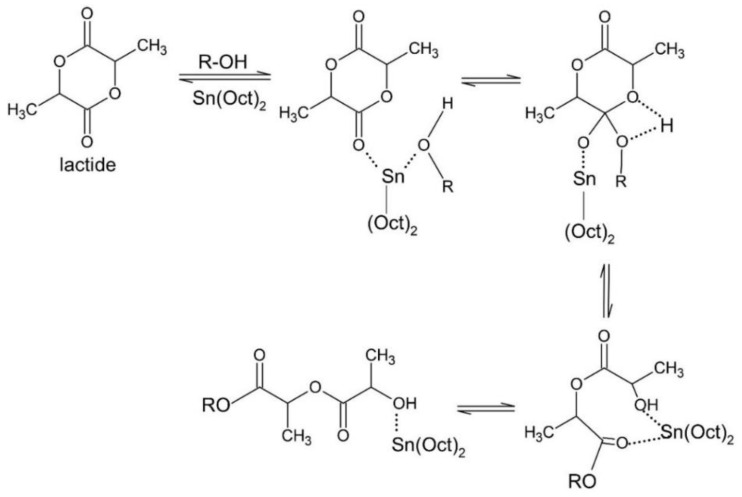
General lactide coordination-insertion mechanism with Sn(Oct)_2_ for PLA production (reprinted with permission [18], Copyright 2000 John Wiley & Sons Inc.).

In the presence of a catalyst, here described as tin octoate (Sn(Oct)_2_), the lactide starts the process by ROP, through an insertion-coordination mechanism that increases the polymer chain, as shown in Figure 4. Such a catalyst is attractive, as the American Food and Drug Administration (USFDA) approved its use for lactide-based polymers as food additive, being suitable for contact with food, as polymeric packaging [17].

**Figure 5:**
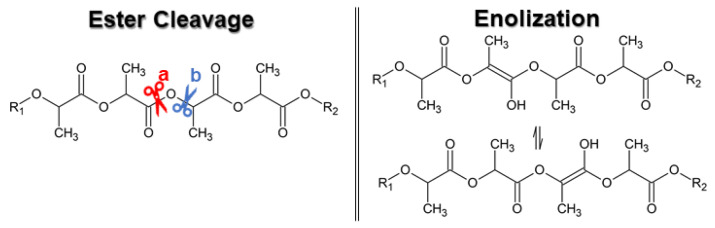
Ester cleavage: (a) carbonyl-oxygen bond, and (b) alkyl-oxygen bond (reprinted with permission [19], Copyright 1997 American Chemical Society); and enolization.

In literature, it is found that the depolymerization of the PLA oligomer is favored at high temperatures and low pressures, through thermal degradation reactions. A higher reaction temperature implies greater racemization and, therefore, lowers the purity of lactide, as presented in studies about the effect of catalysts and polymerization conditions [19]. Racemization occurs due to the cleavage of the ester group at two different points (Figure 5), and only at high temperatures there is a cleavage of the alkyl-oxygen bond. Additionally, the enolization of the ester group also contributes to racemization.

Regarding the use of acidic catalysts, it is described that strong acids (e.g., hydrochloric acid, HCl, or sulfuric acid, H_2_SO_4_) catalyze the carbonyl-oxygen bond cleavage, while Lewis acid-type catalysts promote the alkyl-oxygen bond cleavage [19].

As the molar mass, the crystallinity of the polymer influences its applications. The presence of lactide isomers is responsible for introducing irregularities in the polymer chains, which limits its crystallinity degree as well its application in the medical field. Thus, the cyclic dimer from the levogyre enantiomer of the lactide is the most suitable for applications that require a high degree of crystallinity and mechanical strength [20].

The lactide purity depends on the reactional conditions for lactide synthesis and the prepolymer composition obtained in the polycondensation stage; thus, both steps must be considered to optimize the process parameters. Lactide purification is a crucial step to produce PLA with adequate properties and performance, directly affecting the PLA’s final price. One of the main problems regarding the purity of the product is the racemization of the PLA, which occurs in the production step of the dimer and the PLA itself, which culminates in the production of PLA with inferior properties. Therefore, it is extremely important to understand the operational parameters’ effects on lactide production (yield) and purity (enantioselectivity) [21].

## 3. Properties

**Figure 6:**
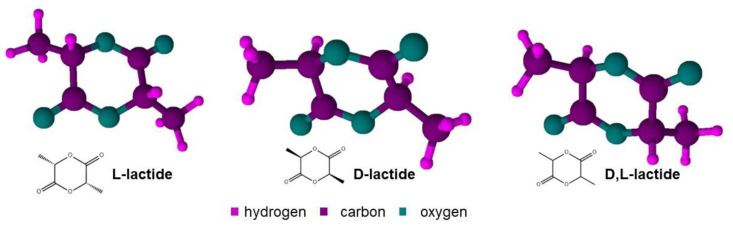
Chemical structure of D-, M-, and L-lactide, respectively (reprinted with permission [12], Copyright 1998 Elsevier).

Lactide has three isomeric forms, (S,S)-L, (R,R)-D, and (S,R)/(R,S)-*meso* (Figure 6), which L-lactide is the most commercially readily available [22,23]. The lactic acid optical activity controls the isomerism of the synthesized lactide, which respectively dictates the properties of the final polymer [24].

The optical components of the lactic acid used in the lactide synthesis regulate its isomerism, which heads the final polymer properties [24]. The physicochemical properties of the lactide do not differ much between its isomeric forms but significantly affect the polymerization reactions in which poly(lactide) with different properties and characteristics are obtained, such as tactile structures, melting point (T_m_), mechanical properties, degradation rate, crystallization rate, and the extent of crystallization, etc. [22,23,24,25,26]. Among these properties, the lactides’ T_m_ is considered one of the most interesting properties for polymer synthesis [23,27], since ROP occurs at temperatures above the lactides’ T_m_ and under the thermal degradation temperature of PLA [6], also interfering with polymer processing [23,27].

The lactides’ T_m_ range from: 95–98 °C, D- and L-lactide [23,27]; 53–54 °C, *meso*-lactide; and, 122–126 °C, *rac*-lactide [23]. Poly(L-lactic acid) (PLLA), for example, has an equilibrium melting point at approximately 207 °C, and practical T_m_ around 180 °C when crystallized at ≈100 °C. Whereas the minimum processing temperature for polymers is usually about 20 °C above the T_m_, the processing temperature for PLLA is up to 200 °C [24]. Therefore, using different isomers for the poly(lactide) (copolymerization) synthesis results in copolymers of lactides with different thermal properties and macromolecular architectures [6,24,28,29]. These differences between the copolymers obtained are dependent on the reactions between the comonomers and the polymerization conditions, affecting the conversion and distribution of comonomers [6,24,28,29]. ROP allows greater control over the distribution of comonomers in the polymer chain [25,28], in addition to low polydispersity, high molar mass, and high levels of end-groups, when compared to polycondensation [28]. This type of polymerization can be performed in solution, bulk, melt, or suspension employing different polymerization mechanisms [29]. When it comes to enantiomerically pure monomers (L-lactide or D-lactide), there is a formation of a polymer chain of isotactic structure; using *rac*-lactide as a monomer can also be used to obtain an “isotatic” structure. Polymers formed by *meso*-lactide monomers can present a syndiotactic structure, with an alternating arrangement of D- and L-lactide isomers, or heterotactic, with a double arrangement of D- and L-lactide isomers [28]. Polymerization of L,L-lactide obtains a linear homopolymer chain architecture with high molar mass (above 70 kDa). The polymerization of L,L-lactide or D,D-lactide with *meso*-lactide gives a linear copolymer macromolecular architecture with high molar mass. *Rac*- and *meso*-lactide makes it possible to obtain linear copolymers with molar mass ranging from low (<20 kDa) to high molar mass (>70 kDa) [29]. In the literature, it is possible to observe the variety of molar mass obtained for these polymers since the use of catalysts, initiators, and different reaction conditions promote considerable differences in the molar mass of the polymers obtained. It is observed that each type of catalyst leads to a certain mechanism of polymerization, which can be ionic, coordination, or free radical [29]. Industrial processes using tin octanoate [Sn(Oct)_2_] as a catalyst of the polymerization reaction, by coordination-insertion mechanism, achieve polymers of molar mass above 100 kDa or even 1000 kDa when performed in the presence of alcohol. Coordination-insertion mechanisms are also explored using other types of catalysts, such as aluminum alkoxides. In this case, polymers of molar mass less than 100 kD are obtained, in reactions that can take days to occur [25]. In addition to these, other catalysts composed of transition metals are presented in the literature [29]. The possibility of using different racemic structures of lactides in polymer synthesis brings a significant benefit to polymer properties, such as the possibility of processing at lower temperatures and lower rates of hydrolytic and oxidative degradation [24]. However, the polymerization of cyclic monomers depends on thermodynamic and kinetic factors [30]. From a thermodynamic point of view, lactide is a singular six-membered ring that is polymerizable [25,30]. It exhibits negative polymerization enthalpy, indicating a high exothermic reaction, around −23 kJ/mol [25] to −27.0 kJ/mol, and entropy of −13.0 kJ/mol·K at 400 K [23]. Regarding solubility, lactide can be soluble in water, alcohols (methanol, isopropanol), oxygenated (ethyl acetate, acetone, butanone, tetrahydrofuran), and organochloride (methylene chloride, chloroform) solvents, besides the organic ones (benzene, toluene, xylene). In water, the lactide hydrolyzes to lactic acid at room temperature. The *meso*-lactide rate of hydrolysis is higher than the D/L-lactide. In other solvents, the lactide solubility is proportional to the subjected temperature [31]. Some properties of lactides found in the literature are presented in Table 2.

In a work carried out for PLGA synthesis by ROP, the characterization of the commercial L-lactide monomer (Purasorb^®^ L) used in the development of the research was carried out [32]. The data obtained showed that L-lactide degrades completely at a temperature of 233 °C observed by thermogravimetric analysis, presenting a single stage of mass loss in the thermal profile. The infrared absorption analysis (FTIR) of the L-lactide shows strong absorption bands characteristics of L-lactide at approximately 935 cm^−1^ (ester group C-COO stretching), 1056 cm^−1^ (COO ring deformation), 1093 cm^−1^ (C-C stretching), 1240 cm^−1^ (symmetric COC lactone ring stretching), 1266 cm^−1^ (CH_2_ and CH wagging), and 1754 cm^−1^ (asymmetric CH_3_ deformation) [13,32,33]. The X-ray diffractogram presented in the same work shows the crystalline microstructure of L-lactide with intense peaks at approximately 2Ɵ equal to 8, 13, and 14° [13,33,34]. Complementing the lactide characterization topic, its nuclear magnetic resonance analysis (^1^H NMR) shows two signals in the L-lactide spectrum, one corresponds to a doublet at δ 1.6 ppm, and the other presents a quadruplet at δ 5.0 ppm, that are respective to the methine (CH) and methyl (CH_3_) groups of the L-lactide molecule [35]. The other lactide isomers present chemical shifts close to the values presented for the L-lactide, with a doublet signal at δ 1.4–1.8 ppm for D- and *meso*-lactides [36]. Some efforts to differentiate and quantify the lactide isomers via chromatographic techniques have been published [37].

## 4. Methods for Lactide Production

Obtaining lactide is an interesting process for high molar mass PLA via ROP, since lactide is the monomer of this polymerization. Few works are found in the literature about the process of obtaining this monomer. Several studies aimed the better comprehension of the experimental conditions, such as temperature, pressure, atmosphere, type, and quantity of catalyst, besides the purification method; on the yield, selectivity, and purity of the lactide produced.

The maximum yield of lactide via polycondensation occurs if the degree of polymerization is equal to 2, according to the patented procedure [38]. In addition, this patent claimed that the production of the dimer occurs through an “end-biting” reaction. They also suggested the recovery of the lactide from the low molar PLA mixture, ignoring the step in which the back-biting reaction occurs. They also pointed out that high temperatures are necessary to distill the lactide to increase viscosity and molar mass.

A patent for the production of lactide via PLA depolymerization by backbiting comprises [39]: (i) thermo-catalytic depolymerization of polylactide to produce lactide in the presence of cocatalyst; (ii) product vaporization occurs in the reaction stage; (iii) product recovery by condensation; (iv) separation of L-, D- or *meso*-lactide.

Metal complexes had been investigated to synthesize the lactide [2,40]. Tin-based catalysts at higher temperature and lower pressure conditions decreased the lactide yield and improved its purity [14]. This occurs because the depolymerization reaction rate is higher in high temperatures and low pressures accelerate the elimination of basic impurities, minimizing the racemization.

Some recognized processes published in the literature for lactide production are summarized in Table 3.

## 5. Lactide Industrial Production

For the current commercial production of lactide, the first step is to remove the water of lactic acid, without the use of solvents, to produce a low molar mass prepolymer (oligomer). The second step is the catalyzed depolymerization of the oligomer, forming the cyclic dimer (lactide), which is purified through distillation [72]. Both steps must be performed at vigorous heating and vacuum to aid the continuous removal of water and lactide. The L-lactide selectivity must be between 60 and 70% to avoid racemization and undesired products. These conditions are highly energy-intensive, requiring strenuous recycling and downstream purification steps, consequently turning this process expensive [44].

**Figure 7:**
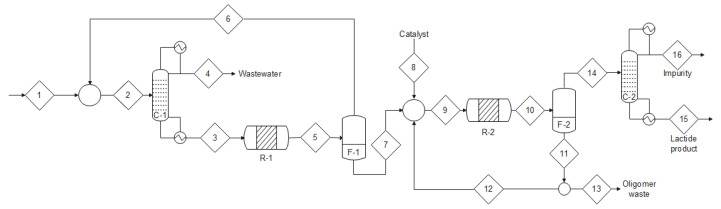
Conventional two-step lactide synthesis process (reprinted with permission [48], Copyright 2019 American Chemical Society): (1) lactic acid stream, (2) lactic acid + reflux mixed stream, (3) dehydrated lactic acid stream, (4) wastewater, (5) dehydrated lactic acid stream, (6) lactic acid recovery, (7) R-1 liquid phase, (8) catalyst feed stream, (9) lactic acid + catalyst mixed stream, (10) oligomers stream, (11) unreacted oligomers stream, (12) unreacted oligomer recovery, (13) high molar mass oligomers, (14) crude lactide stream, (15) purified lactide stream, (16) unpurified lactide stream, (C-1, C-2) distillation columns, (R-1, R-2) polycondensation and depolymerization reactors, respectively, (F-1, F-2) heating duties.

The conventional two-step lactide synthesis process for lactide production on an industrial scale is set up at 200 °C, 5 mmHg, and 0.25 %wt of catalyst (Sn(Oct)_2_) [21], as depicted in Figure 7. In this process, the reactor R-1 promotes lactic acid dehydration and polymerization, operating at low pressure (50–200 mmHg) and high temperature (200 °C), while the oligomers are formed by a self-catalyzed esterification reaction. Since these reactions are reversible, there is an equilibrium among monomers, oligomers, and water. Thus, to increase the conversion of lactic acid, the water vapor is removed from the reactor (stream 6) and fed into the water removal column (C-1) [48].

The lactic acid oligomers formed in R-1 are fed into R-2, where a catalytic depolymerization reaction occurs, producing lactide. This reactor operates at vacuum pressure (10–50 mmHg) and high temperature (200–240 °C) to facilitate the reaction, vaporize and remove the lactide [48]. The unreacted oligomers are recycled (stream 11), and the high molecular weight oligomers are discarded (stream 13). Lastly, the crude lactide goes to the purification column (C-2), where the lactide is purified [48].

**Figure 8:**
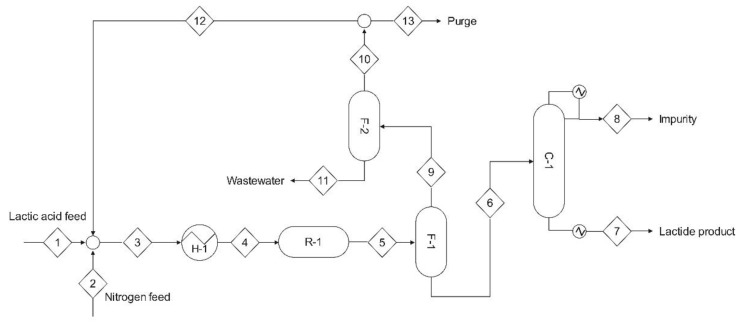
One-step lactide synthesis process (reprinted with permission [48], Copyright 2019 American Chemical Society): (1) lactic acid feed stream, (2) nitrogen stream, (3) lactic acid + N_2_ mixed stream, (4) vaporized mixed stream, (5) reactor outlet, (6) crude lactide stream, (7) purified lactide, (8) unpurified stream, (9) vapor outlet of the F-1 flash drum, (10) gas outlet, (11) waste water, (12) N_2_ gas recycling, (13) N_2_ purged, (R-1) fixed-bed plug flown reactor filled with SiO_2_/Al_2_O_3_, (H-1, F-1, F-2) heating duties, (C-1) distillation column.

A one-step lactide synthesis process (Figure 8) involves lactic acid mixing with nitrogen. To minimize oligomers formation, this stream boils in the preheater, H-1 [48]. Then, it is fed into the reactor, R-1, which is supported with SiO_2_/Al_2_O_3_ catalyst, operates at a high temperature (220–240 °C), and can be operated at atmospheric pressure. The reactor product goes into the first flash drum, F-1, where nitrogen and water are separated from the other compounds, and goes to F-2, where the wastewater is condensed, discarded, while the nitrogen gas is partially recycled and purged. Finally, crude lactide that is left in F-1 is fed into the distillation unity C-1, which produces purified lactide.

Another process that can be used for lactide production is reactive distillation [73]; this is a good alternative since it is a one-step procedure, allowing chemical reaction (lactide synthesis) and physical separation (water removal), reducing cost and increasing productivity. In accord with the literature, the production of lactide via lactic acid distillation is an innovative process, as it is largely used for the purification of lactic acid produced by the fermentative route [74]. The reactive distillation to obtain lactide from the depolymerization of PLA (recycling) uses zinc (II) acetate as a catalyst at 200 °C, producing a lactide with great yield (98%) and purity [75].

**Figure 9:**
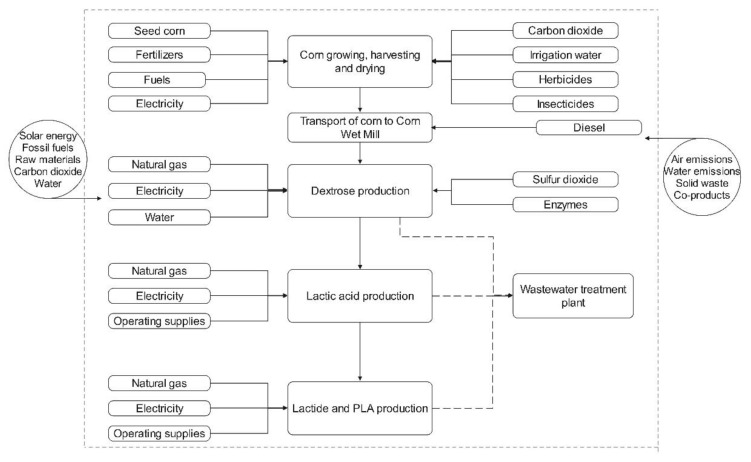
Simplified flow diagram of the life cycle Cargill Dow process for lactide and PLA production (reprinted with permission [72], Copyright 2003 Elsevier).

A simplified flow diagram of the life cycle production of PLA and lactide according to the Cargill Dow concept of sustainability is presented in Figure 9. Its operational supplies refer to chemicals inputs used in the process for controlling the temperature, atmosphere, and the synthesis’ materials in general (raw materials, catalysts, solvents for purification, etc.) [72,76].

**Figure 10:**
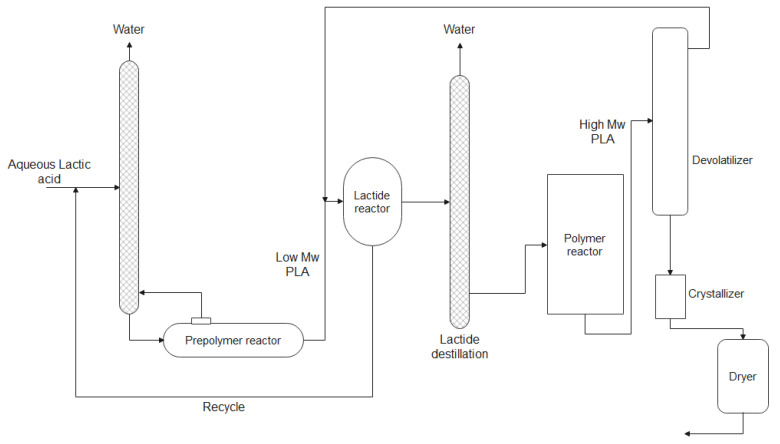
Industrial plant design of Natureworks^TM^ (Cargill Dow) for the production of lactide and PLA (reprinted with permission [12], Copyright 1998 Elsevier).

In another study, it was analyzed the production of lactic acid, lactide, and PLA, using food residue as the input stream; the mass balance proved the viability of the sustainable cycle of lactide production with high yields for the desired products. Additionally, they estimated a minimum selling price for lactide as about $2073 TM [77]. The industrial plant design from Natureworks^TM^ (Cargill Dow) to produce lactide and PLA via a solvent-free process is shown in Figure 10.

## 6. Applications in Bioengineering

Lactide is an intermediate for the production of the high molar mass PLA via ROP. This monomer has great importance since it controls the synthesis for polymer production. The processing, crystallization, and degradation behavior of PLA all depend on the structure and composition of the polymer chains, in particular, the PLA stereochemical structure, which is influenced by the lactide isomers [78]. The presence of L- or D- monomer affects the physical characteristics of the final polymer. PLLA polymer has the highest melting point among the other PLA types due to its crystalline nature. As D-isomer is incorporated in the PLA chain, it reduces its crystallinity, lowering the melting points of PLA copolymers until they become amorphous at D-contents higher than 12% [78]. As a consequence of lactide reactivity, it can also react with other monomers, yielding copolymers with variable properties, as the copolymerization procedure enables the obtention of a final material with target properties in accord to its application [79,80].

The main applications of this cyclic-dimer, lactide, are mainly for the production of the PLA polymer, taking into account which isomer of lactide is part of the synthesis and finally, some copolymers with trimethylene carbonate (TMC) [81], poly(ε-caprolactone) (PCL) [82], polyethylene glycol (PEG) [5], and polyglycolic acid (PGA) [2,3,5,83]. Mainly for medical purposes: tissue engineering, drug delivery, and medical devices [2,3] as described in Table 4.

Polymeric materials based on lactide and other hydroxy acids are widely used in the manufacture of medical products for general and reconstructive surgery, traumatology, and orthopedics, to restore vital human organs and tissues. The main advantage of these materials is biodegradation since the products from their degradation are not injurious to the human body. Thus, it is not necessary for an invasive procedure for implant removal, which is an economical advantage for both patients and physicians [16,84].

PLA is a good candidate for biopolymers to be used in medicine because of its biological, chemical, and physical properties; it has good thermal processability and can be processed by conventional polymer processing methods (injection, extrusion, thermoforming, spinning, etc.), requiring less energy demand when compared to petroleum-based polymers. In addition to that, poly(D-lactide acid) (PDLA) has slower biodegradation than PLLA, and for this reason, PLLA (higher crystallinity) is often more used than PDLA [84,85,86].

**Figure 11:**
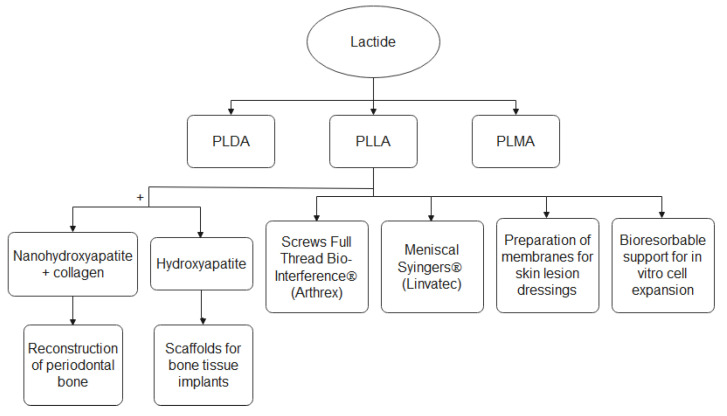
Application of PLA in medicine.

Medical products based on biopolymers assist in dysfunctions and diseases in the musculoskeletal and cardiovascular system, speeding up the tissue healing after an invasive procedure, and preventing post-surgical synechiae [16]. Some of the main polymers starting with lactide, and their medical applications are presented in Figure 11.

Low molar mass PLA has been used for drug delivery as well, for microencapsulation [87], and to release antibiotics against bone infection [88].

Beyond the medical area, PLA is commonly used to produce commodities such as bottles and films for food packaging, being its recyclability code “7” [89]. Moreover, it can partially replace some petroleum plastics, such as polyethylene (PE), polypropylene (PP), polystyrene (PS), and polyethylene terephthalate (PET) [21,90,91]. This biodegradable polymer has increased interest to replace commodity polymers since it is biobased, environmentally friendly, and possible for composting, mechanical, and chemical recycling, which valuate the carbon cycle [92,93].

The extensive application of PLLA highlights the importance of studying the L-lactide dimer since this is the starting monomer to produce this biopolymer.

**Table 4:** Lactide as raw material for polymers production and their applications.

Lactide	Polymers	Applications	References
L-lactide	L-lactide for producing PLLA	Membranes and films for medical applications and 3D printing for prosthesis	[94,95]
D-lactide	D-lactide for producing PDLA	Hydrogel and particles for drug delivery	[96]
L-lactide	L-lactide with PEG	Medical applications, drug vehicles, nanoparticles loaded with bioactive compounds, treatment for cancer and infections	[97,98]
D-lactide	D-lactide with PEG	Biochemical device and packaging	[25]
L-lactide	L-lactide with poly(trimethylene carbonate)	Biodegradable elastomeric scaffold for vascular engineering	[99]
L-lactide	L-lactide with PCL	Absorbable suture medical application due to good tensile properties Packaging application thanks to tunable barrier properties	[100,101,102]
L-lactide/D-lactide	Lactide with lignin	Bio-based composite materials	[103]
L-lactide/DL-lactide	L-lactide with ε-caprolactone and hydroxyapatite	Composite materials for bone reconstruction	[104]
L-lactide	L-lactide with hydroxyapatite	Composite scaffolds for bone tissue engineering	[105]
L-lactide	L-lactide, glycolide, butyl succinate/citrate	Bioabsorbable block copolymers for tissue engineering	[106]
L-lactide	L-lactide with PGA	Smart polymer used as drug delivery device	[83]

## 7. Conclusions

The eco-profile of lactides, biobased and biodegradable, has increased their market interest to produce PLA to minimize the dependence on petroleum-based materials, and to leverage the green economy. Such a sustainable approach can be found in the industrial-scale production of lactide by the Cargill-Dow process. The excellent properties of lactide-based polymers boosted numerous applications that have heated the economic market. There are several works about procedures for lactide synthesis, they have different routes, but primarily the lactic acid dehydration step needs considerable attention from the scientific community. Lactic acid dehydration is primordial since water hydrolysis of the lactide affects the posterior polymerization stage; additionally, it usually employs vacuum at high temperatures for considerable periods, which implies high energy consumption; that said, this step needs optimization. Heterogeneous and homogeneous catalysis, one-step continuous process, gas and/or liquid-liquid phase are some methods currently employed for lactide production. Metallic catalysts can limit the lactide application, i.e., in the medical area, if they cannot be removed. A one-step process is desirable, as stop procedures do not occur in this process, which might length the lactide production. The purification step is also crucial to guarantee the lactide stereochemical purity and impurities removal, being realized by distillation or crystallization. It is urgent to study environmentally-friendly solvents, with the possibility of recovery and reuse, and quicker purification methods to minimize ecological impact.

## Figures and Tables

**Table 1:** Recent price market of L-lactide (3s-cis-3,6-dimethyl-1,4-dioxane-2,5-dione) with 98% of purity.

	L-Lactide			D-Lactide			D,L-Lactide		
Manufacturer	Product Number	Packaging (g)	Price (U$D)	Product Number	Packaging (g)	Price (U$D)	Product Number	Packaging (g)	Price (U$D)
AK Scientific	V2322	500	907.00	2682BB	25	269	V3862	1	14.00
Alfa Aesar	L09031	25	66.40				L09026	10	34.70
Alichem				13076170	25	156.00			
Ambeed				A178884	25	102.00			
American Custom Chemicals Corporation	CCH0039810	25	1212.75	CCH0007427	0.005	502.59			
Apollo scientific	OR933335	25	115.00	OR959522	25	370.00		25	60.00
Arctom							AS027325	25	42.00
Chem-Impex	35186	25	50.40						
Crysdot	CD45000285	25	91.00	CD11297333	100	371.00	CD45000014	500	510.00
Matrix Scientific		25	89.00				172406	25	89.00
Medical Isotopes, Inc.	65805	250	2200.00						
Sigma-Aldrich	367044	25	73.00				303143	25	71.70
SynQuest Laboratories	2H25-1-2T	25	184.00	2H25-1-29	25	592.00	2H25-1-2U	25	96.00
TCI Chemical	L0115	25	55.00				L0091	25	62.00
TRC	L113600	25	150.00	L113605	1	1135.00	L113518	0.5	70.00

**Table 2:** Physical properties of the lactides.

Physical Properties	L-Lactide	D-Lactide	*Meso*-Lactide	*Rac*-Lactide
Molecular weight (g/mol)	144.12	144.12	144.12	
Optical rotation in degrees	−260	+260		
Specific rotation (polarimetry toluene, 25 °C)	(−287)–(−300)°	(+287)–(+300)		(−1)–(+1)°
Appearance (visual test)	White crystals			
Melting point (°C)	95–100	95–100	53–54	122–128
Boiling point (°C)	255			142
Heat of fusion (J/g)	146		118; 128	120–170 (DSC 10 °C/min)
Heat of vaporization (kJ/mol)	63			
Solid density (g/mL)	1.32–1.38		1.32–1.38	
Liquid viscosity (mPas) (110 °C)	2.71			
Liquid viscosity (mPas) (120 °C)	2.23			
Liquid viscosity (mPas) (130 °C)	1.88			

**Table 3:** Chemical processes for lactide production.

Experimental Conditions	Results	References
Optimization of the conditions for lactide synthesis—Temperature: 195–230 °C, Pressure: 25–760 mmHg, Catalysts: SnO, SnCl_2_, Sn(Oct)_2_, Sb_2_O_3_, H_2_SO_4_, Atmosphere: with or without N_2_ flux.	A higher conversion rate was found in the presence of SnCl_2_ and Sn(Oct)_2_, the synthesis rate increased with temperature, despite yielding impurities due to thermal degradation.	[2]
Dehydration step—Temperature: 130–190 °C, Atmosphere: N_2_ flux. Lactide polycondensation: Temperature: 190–210 °C, Pressure: 5–25 mmHg, Catalyst: Sn(Oct)_2_, 0.25 %wt (present or absent).	Lactide: 0.6–2.7 kDa.	[21]
Different metallic catalysts for the PLA depolymerization to obtain lactide—Temperature: 190–245 °C, Pressure: 4 mmHg, Catalysts: Al, Ti, Zn, Zr.	None of these catalysts achieved the lactide yield obtained by using only 0.2 mol% of Sn(Oct)_2_.	[40]
The dehydration step occurs in a short-path distillator—Temperature: 120 °C, Pressure: 37.5 mmHg. Oligomerization step—Temperature: 170 °C, Pressure: 75 mmHg, Catalyst: Sn(Oct)_2_, 1 %wt. Reactive distillation in the short-path distillator—Temperature: 250 °C, Pressure: 3.7 mmHg, Catalyst: Sn(Oct)_2_, 2 %wt. Purification by crystallization. Lactide polymerization—Temperature: 170 °C, Pressure: 760 mmHg, Catalyst: Sn(Oct)_2_, 0.04 %wt.	Purity: 99.9%.	[41]
One-step heterogeneous catalytic process with Bronsted acidic zeolite to produce lactide (zeolite can be regenerated to six reactions consecutive)—Temperature: 150–220 °C, Catalyst: H-beta zeolite, 5 %wt, Lactic acid: 50 %aq.	Yield: 83–97%. Purity: 98%.	[42]
Depolymerization of low molar mass PLA—Temperature: 150–220 °C, Pressure: 2 mmHg, Catalyst: SnCl_2_·2H_2_O/p-toluenesulfonic acid, 0.25 %mol.	Oligomer chain length: 4–5.	[43]
One-step continuous catalytic gas-phase transesterification of alkyl lactates without solvents –Temperature: 220 °C, Catalyst: 5 %wt TiO_2_/SiO_2_, L-methyl lactate: 5.7% in N_2_.	Selectivity ≈ 90%.	[44]
Fast one-step continuous catalytic process (<1 s)—Temperature: 240 °C, Pressure: 760 mmHg, Catalyst: SnO_2_/SiO_2_ nanocomposite (SSO-80), N_2_ as carrier, Lactic acid: 75 %aq. The purification step used ethanol as solvent for lactide crystallization.	Lactide yield: 94%. Enantioselectivity: 99%.	[45]
One-step continuous catalytic process to produce lactide—Temperature: 240 °C, Pressure: 760 mmHg, Catalyst: SnO_2_/SiO_2_ nanocomposite (ϕ ≈ 2 nm), Lactic acid.	Lactide yield: 94%. Enantioselectivity: 99%.	[46]
Lactate transesterification—Temperature: 30–250 °C, Pressure: 1–750 mmHg, Catalyst: Ti-based (0.01–10 %mol), Inert atmosphere: N_2_.	High yield. *meso*-lactide.	[47]
One-step catalytic process in the gas phase to produce lactide—Temperature: 160–240 °C, Pressure: 760 mmHg, Catalyst: SiO_2_/Al_2_O_3_, Lactic acid, Inert atmosphere: N_2_.	Yield: 75–90. Lactide purity: 95–97%.	[48]
Lactic acid dehydration—Temperature: 150–160 °C, Pressure: 110–0.01 mmHg, Inert atmosphere: N_2_, Stirring: 60–70 rpm. Oligomerization step—Cooling the oligomer until room temperature, Catalyst: Zn/Sn (ϕ < 150 μm), 0.1–0.5 %wt. Depolymerization step—Temperature: 160–200 °C, Pressure: 110–0.1 mmHg, Inert atmosphere: N_2_. Purification—Crystallization with toluene and washing with ethyl acetate.	Yield > 99%. Optically pure L/D lactide. Impurities < 0.01 %wt.	[49]
A biogenic guanidine creatine catalyst (human-body metabolite) is used to produce optically pure L/D-lactide in an environmentally-friendly synthesis approach (reactive reduced pressure distillation catalysis). Dehydration and polycondensation—L/D-lactide: 90 %wt, Temperature: 130–170 °C, Pressure: 30–60 mmHg. Oligomerization—Catalyst: Creatinine (1:100 to 1:10,000), Temperature: 150–260 °C, Pressure: 2–15 mmHg. Lactide neutralization—Washing with alkali (1–10 %wt) and deionized water; Drying: vacuum at 20–40 °C for 24–36 h.	Optically pure L/D lactide. Lactide: 0.6–1.5 kDa. Free of toxicity and metals.	[50]
This procedure follows a series of steps to produce optically pure lactide: Melt polymerization of the lactic acid—Temperature: 150–160 °C, Pressure: 100–0.1 mmHg, under stirring. Cooling until room temperature, Catalyst: Sn or Zn: 0.1–0.5 %wt, N_2_ atmosphere. Heating from 160–200 °C, under vacuum (110–0.01 mmHg). The purification step used ethyl acetate as solvent for lactide crystallization.	Yield: 99%. L(+) Lactide optical purity: 100%. Impurities: <0.01%.	[51]
One-step heterogeneous catalytic process with zeolite to produce lactide—Temperature: 110–165 °C, Pressure: 760 mmHg, Catalyst: Sn-beta zeolite, SnO_2_-SiO_2_ xerogel, supported SnO_2_/Si-beta, Sn-MCM-41 zeolite, Lactic acid: 50 %aq.	Yield: 88.2–95.8%. Pure L(+) lactide.	[52]
A continuous process to produce lactide from lactic acid in ionic solvent, reducing the temperature and moisture generated during the reaction, preventing the lactide degradation. Temperature: 120–300 °C, Pressure: 1–500 mmHg, Time: 1–5 h.	Yield ≈ 85%.	[53]
Single-step lactide production from aqueous lactic acid in the presence of a solid catalyst (Sn, Pb or their mixture)—Temperature: 170–250 °C, Pressure: 760 mmHg or vacuum, Atmosphere: N_2_, Time: until 100 h.	Conversion rate ≈ 80%. Selectivity ≈ 90%.	[54]
Lactide production through liquid phase depolymerization reaction of lactic acid with tin-derivatives—Temperature: 190–210 °C, Pressure: 760 mmHg, Catalyst: tin (IV) compounds, Additives: di- and trialkylphenols, 0.001–0.1 %wt, Time: 4.5 h.	Yield ≈ 72%.	[55]
Lactide production via lactic acid ester dealcoholization—Temperature: 120–230 °C, Pressure: 0.3–700 mmHg, Catalyst: monobutyl tin, Time: 3.5 h.	Purity ≈ 99.4%. Impurities: acids and moisture.	[56]
Rapid production of lactide from lactic acid or ammonium lactate—Temperature: 180 °C, Pressure: 30 mmHg, Time: 4 h.	Yield ≈ 94%.	[57]
Temperature: 200–250 °C, Pressure: 0.5–10 mmHg, Time: 3 h.	Yield: 64%. Impurities: low content.	[58]
Lactide production through butyl lactate—Temperature: 180 °C, Pressure: 0.7 mmHg, Catalyst: dibutyltin dichloride.	Dehydrated lactide with low hygroscopicity.	[59]
Lactide production from aqueous lactic acid, vaporized and transported with N_2_ to feed the reactor—Temperature: 150–225 °C, Catalyst: Al_2_O_3_.	Lactide purity: 92%. The ratio between the yield of lactide obtained and the molar percentage of lactic acid is 9.1:1.	[60]
Temperature: 130–230 °C, Pressure: 13–25 mmHg, Catalyst: carboxylic acid tin-derivative (≤20 carbon atoms), Lactic acid.	Yield ≤ 80%. Optical purity: 99%.	[61]
Preliminary study for lactide production under different reactional system configurations –Temperature: 185 °C, Pressure: 125 mmHg, Catalyst: Sn(Oct)_2_, 1 %wt, Time: 4 h.	Yield ≈ 8%.	[33]
Lactide purification method to obtain highly optical pure DL-lactide via *meso*-lactide hydrolysis followed by its removal—Dehydration and polycondensation step: Temperature: 180–230 °C, Pressure: 20 mmHg, Catalyst: SnO. The lactide vapor is distilled and condensed at 60–90 °C.	Yield (D/L-lactide) ≈ 55%.	[62]
Lactide production via lactide oligomer depolymerization using microwave irradiation—Temperature: 180 °C, Pressure: 25 mmHg, Lactic acid: 90 %aq, Time: 12 h, Irradiation: 2.45 GHz.	2.7 times more lactide was obtained with microwave irradiation process in comparison to the method under conventional heating.	[63]
Obtaining enantiomerically pure lactides through PLA-oligomer depolymerization using a green and non-toxic catalyst. Temperature: 220 °C, Pressure: 3 mmHg, Catalyst: biogenic creatinine, Time: 2 h.	Yield: 68.5–69.5%.	[64]
Heterogeneous catalysis using zeolite (ZSM-5) to obtain L-lactide—Temperature: 144 °C, L-Lactic acid: 98 %aq + solvent mixture: water and o-xylene, Catalyst: Zeolite ZSM-5, Time: 4 h.	Yield ≈ 89%.	[65]
Study of the lactide’ synthesis from lactic exploring different metallic catalysts—Temperature: 200–250 °C, Pressure: 1–2 mmHg, Catalyst: ZnO, (C_2_H_5_)_2_Mg, Sn(Oct)_2_, L-lactic acid: 85 %aq, Time: 2–30 min	Yield: 27–82%.	[66]
L-lactic acid polycondensation—Temperature: 120–200 °C, Time: 9 h. PLA-oligomer depolymerization—Temperature: 210 °C, Pressure: 76 mmHg, Time: 3 h.	Yield: 38.5%.	[36]
L-lactide production via PLLA thermal depolymerization in a closed system—Temperature: 250–290 °C, Pressure: 3 mmHg, Time: 10 h.	Yield: 8–14%.	[67]
Lactide synthesis from alkyl lactate—Polycondensation—Temperature: 150–180 °C, Pressure: 10–720 mmHg, Atmosphere: N_2_, Alkyl lactate, Time: ≈ 24 h. Depolymerization—Temperature: 180–210 °C, Pressure: 10 mmHg, Catalyst: SnO, 0.5 %wt, Time: 5 h.	Yield: 82%.	[68]
D-lactide synthesis from D-lactic acid—Depolymerization process—Temperature: 230–240 °C, Pressure: 10–200 mmHg, Catalyst: ZnO, 0.01–1.5 %wt, D-lactic acid.	Yield: 65–72%.	[69]
Production of D,L-lactide from D,L-lactic acid with ZnCl_2_ and Cat-A under microwave irradiation: Pressure: 7.5–37.5 mmHg, Time: 1 h, Irradiation: 2.45 GHz.	Yield (D,L-lactide): 36%. Pure D,L-lactide.	[70]
Polycondensation step—Temperature: 150 °C, Pressure: 30 mmHg, Lactic acid, 92 %aq, Atmosphere: N_2_, Time: 5 h. Depolymerization step—Temperature: 130–195 °C, Pressure: 3 mmHg, Catalyst: Zn(la)_2_, NaHCO_3_, Time: 3–5 h.	Yield: 95.6%. Purity: 97.9%.	[71]

## Data Availability

Not applicable.
